# Supplementary material for: WordCommentsAnalyzer: A windows software tool for qualitative research
Source: F1000Res. 2018 Sep 4;7:536. Originally published 2018 May 3. [Version 2] doi: 10.12688/f1000research.14819.2 (PMC6137411; doi:10.12688/f1000research.14819.2)
Supplement: Supplementary file 3 [file f1000research-7-17614-s0002.tgz › b2a79496-4ada-47c4-9682-a99ce867836d.zip!/Eight Iranian Health Journals/Journal of Research in Rehabilitation Sciences _3.docx]

© Journal of Research in Rehabilitation Sciences . This work is licensed under CC BY-NC 4.0 (https://creativecommons.org/licenses/by-nc/4.0/).

Volume & Issue: دوره 13، شماره 2: 1396

Title: تأثیر دستکاری قیود تکلیف بر یادگیری مهارت حرکتی در کودکان

http://jrrs.mui.ac.ir/index.php/jrrs/article/view/2851

مقدمه: با توجه به این‎ که اکتساب مهارت به ‌وسیله قیود تحت تأثیر قرار می‎گیرد، پژوهش حاضر با هدف بررسی تأثیر تمرین با تجهیزات مقیاس‌بندی شده و استاندارد بر یادگیری مهارت پرتاب آزاد بسکتبال در کودکان انجام گردید. مواد و روش‌ها: این مطالعه از نوع نیمه تجربی همراه با مراحل پیش‌آزمون و پس‌آزمون بود. 20 دانش‎آموز دوره ابتدایی 12-10 ساله به ‏روش نمونه‎گیری در دسترس انتخاب شدند و به صورت تصادفی در دو گروه تمرين با تجهیزات مقیاس‎بندی ‎شده و استاندارد قرار گرفتند. مرحله اکتساب، 5 جلسه و در هر جلسه 50 کوشش انجام شد. آزمون‏ها با توپ‌های اندازه 5، 6 و 7 به ترتیب با ارتفاع حلقه 60/2، 80/2 و 05/3 متری انجام گرفت. آزمون انتقال با توپ اندازه 6 در آخرین روز اکتساب و 10 ‎دقیقه پس از آخرین کوشش و آزمون یادداری نیز 24 ساعت بعد از آخرین جلسه اکتساب صورت گرفت. داده‏ها با استفاده از آزمون‏های تحلیل ‏واریانس‏ مرکب، Independent t و Paired t در سطح معنی‏داری 05/0 تجزیه و تحلیل گردید. یافته‌ها: بر اساس مقایسه دقت گروه‏ها در آزمون یادداری و انتقال، تفاوت معنی‏داری بین گروه‏ها وجود نداشت (05/0 < P). با این وجود، الگوی پرتاب گروه‎ تمرین با تجهیزات مقیاس‏بندی ‏شده بهتر از گروه‎ استاندارد بود. نتیجه‎گیری: استفاده از تجهیزات مقیاس‏بندی ‎شده، باعث شکل‎گیری بهتر الگوی حرکت در کودکان می‏شود.

Title: روان‌سنجی مقیاس افسردگی Cornell در سالمندان مبتلا به دمانس شهراصفهان

http://jrrs.mui.ac.ir/index.php/jrrs/article/view/2810

مقدمه: افسردگی، دومین اختلال روان‌شناختی شایع در سالمندان محسوب می‌شود. مطالعه حاضر با هدف روان‌سنجی مقیاس افسردگی Cornell در دمانس (Cornell Scale for Depression in Dementia یا CSDD) در سالمندان مبتلا به دمانس شهر اصفهان انجام گرفت. مواد و روش‌ها: این مطالعه از نوع سایکومتریک بود. 110 بیمار مبتلا به دمانس که بر اساس تشخیص روان‌پزشک و معیارهای Diagnostic and Statistical Manual of Mental Disorders-4th Edition-Text Revision (DSM-IV-TR) به کلینیک سالمندان اصفهان مراجعه کرده بودند، با استفاده از نمونه‌گیری تصادفی انتخاب شدند و مقیاس‌های‌ افسردگی سالمندان (Geriatric Depression Scale یا GDS) و ارزیابی عملکرد شناختی کوتاه (Mini-Mental State Examination یا MMSE) را تکمیل نمودند. سپس داده‌ها با استفاده از آمار توصیفی، تحلیل عاملی، ضریب Cronbach¢s alpha و ضریب همبستگی Pearson در نرم‌افزار SPSS مورد تجزیه و تحلیل قرار گرفت. یافته‌ها: پایایی کل مقیاس 85/0 و پایایی عامل‌های افسردگی، علایم افسردگی سایکوتیک، اختلالات خواب، علایم جسمی و اضطراب به ترتیب 77/0، 63/0، 79/0، 62/0 و 47/0 به دست آمد. با استفاده از ضریب همبستگی Pearson، ضریب همبستگی GDS و MMSE با کل مقیاس CSDD به ترتیب ۴۷/0 (01/0 > P) و 35/0 (01/0 > P) محاسبه گردید. با استفاده ازتحلیل عاملی اکتشافی، پنج عامل به دست آمده فوق دارای واریانس مشترک 72/58 درصد بود. نتیجه‎گیری: نسخه فارسی مقیاس CSDD در جامعه سالمندان مبتلا به دمانس ایران، از خصوصیات روان‌سنجی قابل قبولی برخوردار می‌باشد.

Title: تأثیر دستورالعمل‌های توجهی بر عملکرد تعادلی کودکان عادی و مبتلا به اختلال هماهنگی رشدی (DCD)

http://jrrs.mui.ac.ir/index.php/jrrs/article/view/2871

مقدمه: تحقیقات نشان داده‌اند که برای اکتساب مهارت حرکتی، هدایت کانون توجه بزرگسالان به نشانه‌های خارجی مؤثرتر از نشانه‌های درونی است. مطالعه حاضر با هدف بررسی دستورالعمل‌های توجهی بر عملکرد تعادلی کودکان عادی و کودکان مبتلا به اختلال هماهنگی رشدی (Developmental coordination disorder یا DCD) انجام شد. مواد و روش‌ها: 20 دانشجوی مرد، 20 کودک پسر عادی و 20 کودک پسر مبتلا به DCD با استفاده از پرسش‌نامه مشاهده حرکتی آموزگاران انتخاب شدند. تکلیف، آزمون تعادلی Stork بر روی دیسک بادی بود که تحت دو شرایط آزمایشی شامل کانون توجه درونی (توجه به حداقل‌سازی حرکات پا) و بیرونی (توجه به حداقل‌سازی حرکات دیسک) مورد سنجش قرار گرفت. داده‌ها با استفاده از آزمون‌های ANOVA، Tukey و Paired t تجزیه و تحلیل گردید. یافته‌ها: مطابق با فرضیه عمل محدود شده، بزرگسالان در حالت توجه بیرونی عملکرد بهتری داشتند، اما برای کودکان عادی، به کارگیری کانون توجه درونی مفیدتر بود. تفاوت معنی‌داری بین دو حالت کانون توجه در کودکان مبتلا به DCD مشاهده نشد. نتیجه‎گیری: پیشنهاد می‌شود کاردرمانگران و مربیان ورزشی روش‌های توان‌بخشی و دستورالعمل‌های آموزشی خود را بر اساس سن، تجربه و وضعیت رشدی مراجعان تنظیم نمایند.

Title: تأثیر تمرینات ترکیبی ثبات مرکزی و عصبی- عضلانی بر کنترل پاسچر دانش‌آموزان دارای ناشنوایی مادرزادی

http://jrrs.mui.ac.ir/index.php/jrrs/article/view/2846

مقدمه: نتایج پژوهش‌های انجام شده نشان می‌دهد که ضعف در حفظ تعادل، از جمله مشکلات اصلی در افراد دارای ناشنوایی مادرزادی است و تعادل نیز عامل مهم و مؤثری بر کیفیت زندگی می‌باشد. تقویت و بهبود عملکردهای تعادلی جهت کاهش مشکلات این گروه از افراد جامعه و نزدیک کردن کیفیت زندگی آن‌ها به حالت عادی، باید مورد تأکید قرار گیرد. هدف از انجام مطالعه حاضر، بررسی تأثیر یک دوره تمرینات ترکیبی ثبات مرکزی و عصبی- عضلانی بر کنترل پاسچر دانش‌آموزان دارای ناشنوایی مادرزادی بود. مواد و روش‌ها: این تحقیق از نوع نیمه تجربی با طرح پیش‌آزمون و پس‌آزمون بود که بر روی ۲۴ دانش‌آموز دارای ناشنوایی مادرزادی صورت گرفت. بدین ترتیب، نمونه‌ها به ‌صورت غیر تصادفی هدف‌دار به دو گروه تجربی و شاهد تقسیم شدند (هر گروه ۱۲ نفر). گروه شاهد به زندگی روزمره خود ادامه دادند؛ در حالی‌ که گروه تجربی به مدت شش هفته و به‌ صورت سه جلسه ۶۰ دقیقه‌ای، به تمرینات ترکیبی ثبات مرکزی و عصبی- عضلانی پرداختند. توانایی آزمودنی‌ها در حفظ وضعیت ایستاده در حالت‌های حسی مختلف اندازه‌گیری گردید. میزان تغییرات در حفظ وضعیت ایستاده در حالت‌های حسی مختلف قبل و بعد از شش هفته تمرینات نیز مورد سنجش قرار گرفت. داده‌ها با استفاده از آزمون Paired t در نرم‌افزار SPSS تجزیه و تحلیل شد (05/۰ ≥ P). یافته‌ها: بر اساس نتایج آزمون Paired t، کاهش معنی‌داری در تعداد خطا حالت اول بدون هیچ‌گونه تداخل حسی (009/0 = P)، تعداد خطا حالت دوم و غالب بودن سیستم بینایی (002/0 = P)، تعداد خطا حالت سوم و غالب بودن سیستم حس پیکری (001/0 = P) و تعداد خطا حالت چهارم و غالب بودن سیستم دهلیزی (001/0 = P) بعد از شش هفته مشاهده گردید، اما کاهش معنی‌داری در هیچ‌ یک از حالت‌های حفظ تعادل در گروه شاهد وجود نداشت. نتیجه‎گیری: به نظر می‌رسد تمرینات ترکیبی ثبات مرکزی و عصبی- عضلانی، می‌تواند باعث بهبود وضعیت تعادل ظاهری در معلولان شنوایی شود و می‌توان از آن در کنار سایر برنامه‌های تمرینی استفاده کرد. با این ‌حال، پژوهش حاضر محدودیت‌هایی داشت و انجام تحقیقات بیشتر در این زمینه ضروری می‌باشد.

Title: تأثیر تقویت عضلات دور کننده ران و چرخاننده خارج ران بر آسیب اسپرین خارجی غیر برخوردی مچ پا در فوتبالیست‌ها و فوتسالیست‌های مرد: تحقیق تجربی کنترل شده

http://jrrs.mui.ac.ir/index.php/jrrs/article/view/2856

مقدمه: یکی از رایج‌ترین آسیب‌های ورزشی، آسیب‌های غیر‌ برخوردی مچ پا می‌باشد. ضعف عضلات دور کننده و چرخاننده خارج ران، موجب تغییر راستای زانو و مچ پا می‌شود و می‌تواند با عدم امکان ایجاد تعادل در فرودها، خطر اسپرین‌های غیر برخوردی مچ پا را افزایش دهد. پژوهش حاضر با هدف تعیین اثر تقویت عضلات دور کننده و چرخاننده خارج ران در بروز آسیب‌های غیر برخوردی اسپرین خارجی مچ پا انجام گردید. مواد و روش‌ها: در این مطالعه تجربی کنترل شده، 200 فوتبالیست و فوتسالیست انتخاب شدند و به روش تصادفی در دو گروه تجربی و شاهد قرار گرفتند. پس از اطمینان از همگن بودن گروه‌ها و اندازه‌گیری قدرت عضلات دور کننده و چرخاننده خارج ران، هر دو گروه وارد برنامه بدنسازی شدند. مدت برنامه برای هر دو گروه، 4 جلسه در هفته (هر جلسه یک ساعت) بود و در هر جلسه پس از 45 دقیقه تمرینات مشترک، گروه شاهد برنامه معمول بدنسازی را ادامه می‌داد و گروه تجربی به مدت 15 دقیقه برنامه تمرینات منتخب را اجرا می‌نمود. در پایان 8 هفته، قدرت عضلات دور کننده و چرخاننده خارج ران مجدد اندازه‌گیری و آسیب‌های غیر برخوردی اسپرین خارجی مچ پا در طول یک فصل نیز ثبت گردید. یافته‌ها: 8 هفته برنامه تقویتی منتخب عضلات دور کننده و چرخاننده خارج ران، به طور معنی‌داری باعث افزایش قدرت این عضلات در گروه تجربی شد و در مقایسه با گروه شاهد، منجر به کاهش 1/46 درصدی آسیب غیر برخوردی اسپرین خارجی مچ پا گردید (کاهش از 13 آسیب در گروه شاهد به 7 آسیب در گروه تجربی). نتیجه‎گیری: تقویت عضلات دور کننده و چرخاننده خارج ران با اجرای برنامه منتخب تقویت این عضلات، علاوه بر برنامه‌های بدنسازی معمول، ممکن است موجب کاهش آسیب غیر برخوردی اسپرین خارجی مچ پا گردد.

Title: اثربخشی آموزش قصه‌های مبتنی بر دیالوگ بر مهارت‌های زبانی کودکان دختر کم‌توان هوشی آموزش‌پذیر مقطع دبستان

http://jrrs.mui.ac.ir/index.php/jrrs/article/view/2852

مقدمه: از مهم‌ترین مشکلات کودکان کم‌توان هوشی، محدودیت در مهارت‌های زبانی است. یکی از روش‌های پیشنهاد شده جهت ارتقای مهارت‌های زبانی، استفاده از قصه‌گویی به روش دیالوگ می‌باشد. هدف از انجام پژوهش حاضر، بررسی اثربخشی آموزش قصه‌های مبتنی بر دیالوگ بر مهارت‌های زبانی دختران کم‌توان هوشی مقطع دبستان شهر اصفهان بود. مواد و روش‌ها: این مطالعه از نوع نیمه آزمایشی با طرح پیش‌آزمون- پس‌آزمون همراه با گروه شاهد بود. جهت انتخاب نمونه‌ها، از روش نمونه‌گیری هدفمند استفاده گردید. بدین ترتیب، 30 نفر از دختران کم‌توان هوشی آموزش‌پذیر در مقطع دبستان انتخاب شدند و به روش تصادفی ساده در دو گروه شاهد و آزمایش قرار گرفتند. گروه آزمایش به مدت 8 جلسه، آموزش قصه‌های دیالوگی مبتنی بر تکنیک‌های Whitehurst را دریافت کردند. مهارت‌های زبانی کودکان با استفاده از نسخه اولیه تجدید نظر شده آزمون رشد زبان- ویرایش سوم (Test of language development-Primary:3rd Edition یا TOLD-P:3) مورد سنجش قرار گرفت. داده‌های به دست آمده با روش تحلیل کواریانس در نرم‌افزار SPSS و در سطح معنی‌داری 01/0 > P تجزیه و تحلیل گردید. یافته‌ها: تفاوت معنی‌داری بين دو گروه آزمايش و شاهد در پس‌آزمون با کسر اثر پيش‌آزمون، در مؤلفه‌هاي زبان گفتاری (010/0 > P، 58/155 = F)، گوش کردن (010/0 > P، 71/104 = F)، سازماندهی (010/0 > P، 40/76 = F)، صحبت کردن (010/0 > P، 19/102 = F)، معنا‌شناسی (010/0 > P، 07/82 = F)، نحو (010/0 > P، 07/82 = F) و نمره کل مهارت زبانی (010/0 > P ،427 = F) مشاهده شد. نتیجه‎گیری: قصه خواندن به روش دیالوگ به صورت تعاملات گروهی برای کودکان کم‌توان هوشی، زمینه‌ای برای فراگیری زبان می‌باشد. همچنین، کودکان طی مشارکت و تعامل حین قصه‌گویی، تکرار، تمرین، دریافت بازخورد و تقویت، می‌توانند مهارت‌های زبانی خود را بهبود بخشند.

Title: تأثیر درمان شناختی بر کاهش افسردگی بزرگسالان دارای لکنت زبان

http://jrrs.mui.ac.ir/index.php/jrrs/article/view/2875

مقدمه: هدف از انجام پژوهش حاضر، تعیین اثربخشی درمان شناختی بر کاهش افسردگی بزرگسالان دارای لکنت بود. مواد و روش‌ها: این مطالعه به روش نیمه آزمایشی و همراه با طرح پیش‌آزمون، پس‌آزمون و گروه شاهد انجام شد. جامعه آماری پژوهش شامل کلیه بزرگسالان مراجعه کننده به کلینیک‌های گفتار درمانی شهر کرج در سال 1395 بود. 40 نفر از مراجعه کنندگان مبتلا به افسردگی، به صورت تصادفی در دو گروه آزمایش و شاهد قرار گرفتند و سطح افسردگی همه آزمودنی‌ها با استفاده از مقیاس افسردگی Beck (Beck Depression Inventory یا BDI) اندازه‌گیری گردید (پیش‌آزمون). گروه آزمایش درمان شناختی (Cognitive therapy یا CT) را به صورت گروهی در 10 جلسه 120 دقیقه‌ای دریافت کردند. در پایان مداخلات، سطح افسردگی همه آزمودنی‌ها مجدد اندازه‌گیری شد (پس‌آزمون). داده‌ها با استفاده از تحلیل کواریانس در نرم‌افزار SPSS مورد تجزیه و تحلیل قرار گرفت. یافته‌ها: مقایسه میانگین نمرات پیش‌آزمون و پس‌آزمون در دو گروه نشان داد که میانگین نمرات پس‌آزمون افسردگی در گروه آزمایش کاهش یافت (010/0 > P). نتیجه‎گیری: رویکرد CT روش بسیار مناسبی جهت کاهش افسردگی بزرگسالان دارای لکنت می‌باشد. همچنین، با توجه به اثربخش بودن مداخله، می‌توان از این برنامه به طور گسترده برای درمان اختلالات روانی این دسته از افراد در کلینیک‌های درمانی استفاده کرد.

Title: مقایسه علایم مقیاس ناراحتی مسیر صوتی (VTD) و ویژگی‌های آکوستیک در بیماران مبتلا به ریفلاکس حلقی- حنجره‌ای (LPR) و ریفلاکس معدی- مروی (GERD)

http://jrrs.mui.ac.ir/index.php/jrrs/article/view/2822

مقدمه: بیماری‌های ریفلاکس معدی- مروی (Gastro esophageal reflux disease یا GERD) و ریفلاکس حلقی- حنجره‌ای (Laryngopharyngeal reflux یا LPR) از جمله بیماری‌های مرتبط با اختلالات صوتی می‌باشند که موجب تغییراتی در تارآواها و به دنبال آن، تغییر در کیفیت صوتی و احساسات ناخوشایند در مسیر صوتی می‌شود. با توجه به این که LPR نسبت به GERD تأثیر شدیدتر و مستقیم‌تری بر روی مخاط حلقی- حنجره‌ای و تارآواها دارد، انتظار می‌رود علایم حسی و آکوستیکی پایدارتری نسبت به GERD ایجاد کند. بنابراین، مطالعه حاضر با هدف مقایسه افراد مبتلا به LPR و GERD و بررسی ویژگی‌های آکوستیک و علایم ناراحتی مسیر صوتی آن‌ها انجام شد. مواد و روش‌ها: این پژوهش از نوع مقطعی بود و به روش توصیفی- تحلیلی انجام گرفت. نمونه‌های مطالعه را ۱۷ بیمار مبتلا به GERD و ۱۸ بیمار مبتلا به LPR تشکیل داد. صدای بیماران در اتاق آکوستیک (نویز 28 دسی‌بل) ضبط شد و سپس مقیاس ناراحتی مسیر صوتی (Vocal Tract Discomfort یا VTD) توسط هر دو گروه از بیماران تکمیل گردید. ویژگی‌های آکوستیکی بیماران در نرم‌افزار Praat آنالیز شد و مقادیر به دست آمده و همچنین، نمرات شدت و تکرار VTD در نرم‌افزار SPSS مورد تجزیه و تحلیل قرار گرفت. یافته‌ها: تفاوت معنی‌داری بین ویژگی‌های فرکانس پایه، آشفتگی دامنه (لوکال)، آشفتگی فرکانس (لوکال) و نسبت هارمونی به نویز دو گروه وجود نداشت (050/0 < P). در علایم VTD نیز تفاوت معنی‌داری بین متغیرهای تکرار و شدت درد و تکرار التهاب دو گروه مشاهده شد (050/0 > P). نتیجه‎گیری: تکرار و شدت درد و تکرار التهاب در افراد مبتلا به LPR نسبت به GERD بیشتر می‌باشد. می‌توان نتیجه گرفت که بیماری LPR به دلیل درگیر کردن فضای حلقی- حنجره‌ای، تأثیر مخرب‌تری بر مسیر صوتی و ویژگی‌های آکوستیکی افراد مبتلا می‌گذارد؛ در صورتی که GERD تأثیر مستقیمی بر این نواحی ندارد.
